# The Efficiency of Primary Health Care Institutions in the Counties of Hunan Province, China: Data from 2009 to 2017

**DOI:** 10.3390/ijerph17051781

**Published:** 2020-03-09

**Authors:** Kaili Zhong, Lv Chen, Sixiang Cheng, Hongjun Chen, Fei Long

**Affiliations:** 1 Department of Social Medicine and Health Management, Xiangya School of Public Health, Central South University, Changsha 410078, China; 176911069@csu.edu.cn (K.Z.); csxaai@163.com (S.C.); 2 Department of Primary Health Care, Health Commission of Hunan Province, Changsha 410078, China; zngw123@163.com (H.C.); zkellly@163.com (F.L.)

**Keywords:** primary health care, data envelopment analysis, Malmquist index model, universal health coverage

## Abstract

This study aimed to estimate the efficiency and its influencing factors of Primary Health Care Institutions (PHCIs) in counties in Hunan Province, China, and put forward feasible suggestions for improving the efficiency of PHCIs in Hunan Province. We applied the Input-Oriented Data Envelopment Analysis (DEA) method and the Malmquist Index Model to estimate the efficiency of PHCIs in 86 counties in Hunan Province from 2009 to 2017. Then, the Tobit model was used to estimate the factors that influence the efficiency of PHCIs. Since the implementation of the new health-care reform in 2009, the number of health resources in PHCIs in Hunan Province has increased significantly, but most counties’ PHCIs remain inefficient. The efficiency of PHCIs is mainly affected by the total population, city level, the proportion of health technicians and the proportion of beds, but the changes in per capita GDP have not yet played a significant role in influencing efficiency. In the future, the efficiency of PHCIs should be improved by increasing medical technology skills and enthusiasm of health technicians and by improving the payment policies of medical insurance funds.

## 1. Introduction

Achieving universal health coverage (UHC) is one of the targets of the World Health Organization (WHO). Primary Health Care (PHC) can meet the majority of a person’s health needs over the course of their life and health systems with strong PHC are needed to achieve UHC [1]. In China, Primary Health Care Institutions (PHCIs), which are the providers of PHC, not only undertake general disease diagnosis and treatment but also undertake basic public health services [2]. The current hospital-centered health service system consumes a large number of health funds, but it cannot meet the ever-changing needs of people and does not have long-term sustainability [3]. Compared with hospitals, PHCIs are more suitable for the prevention and control of major diseases, reducing the incidence of diseases at the source and improving the health of residents. PHCIs in China consist of community health service centers, community health service stations, township health centers and village clinics [4]. Since the new health-care reform, initiated in 2009, China has introduced a series of policy measures in the areas of medical insurance policies, hierarchical medical systems and general practitioner systems to improve the quality of PHCIs’ services and guide residents to first visit PHCIs [5,6,7].

In the past nine years, the new health-care reform has made some progress, but there are also some problems [8]. According to the Statistical Communique on the Development of China’s Health Care Industry, the number of beds and the number of health technicians in PHCIs have increased by 38.98% and 36.66% from 2009 to 2017, respectively. However, their proportion of all medical institutions has decreased by 5.65% and 5.25%, respectively. Meanwhile, the number of outpatients/inpatients in PHCIs accounted for 61.75%/31.01% of the total outpatients/inpatients in 2009, yet decreased to 54.16%/18.21% in 2017 [9,10]. Affected by factors such as geography, the economy and policies, the provision and utilization of PHC in China varies from region to region, and between urban and rural areas [11,12,13]. With the construction of China’s county medical alliances, it is extremely important to improve the efficiency of PHCIs in the counties. Therefore, it is necessary to measure the efficiency of PHCIs, which could provide a policy basis for the health administration departments, and better guide the distribution and utilization of health resources.

Data Envelopment Analysis (DEA) is a non-parametric approach that uses linear programming to build a piece-wise linear-segmentation efficiency frontier based on best practice [14]. DEA has become an effective tool for measuring the efficiency of health care since the mid-1980s, and has been widely used in PHC over the past two decades [15,16]. Scholars usually evaluate PHC from two perspectives—institutions and regions—and some scholars evaluate the specific services, such as oral health care in PHC [17,18,19]. In China, studies from the perspectives of institutions and provinces have shown that the efficiency of PHCIs is low [20,21,22,23]. However, there are few studies that have evaluated the efficiency of PHCIs from the perspective of counties.

In this paper, we focused on the efficiency of PHCIs in 86 counties in Hunan Province, China. Hunan Province is located in central China and has 18 county-level cities, 61 counties, seven autonomous counties and 36 municipal districts. Its population was 68.89 million in 2018, of which the urban population accounts for 56.02% [24]. In recent years, Hunan Province has been committed to the construction of PHCIs to meet the health needs of the residents. While much has changed in Hunan’s PHCIs, little is known about the efficiency. This article studies the service efficiency and its influencing factors of PHCIs in Hunan Province from 2009 to 2017 in units of counties, and puts forward feasible suggestions for improving the efficiency of PHCIs in Hunan Province. The main objectives of this paper were: (1) To measure the efficiency of Hunan’s PHCIs during the new health-care reform from 2009 to 2017; (2) to evaluate changes in productivity during the new health-care reform; (3) to determine the influencing factors of Technical Efficiency (TE); and (4) to make feasible suggestions for improving the efficiency of PHCIs.

## 2. Materials and Methods 

### 2.1. DEA

DEA is a nonparametric method for examining the relative efficiency of similar decision-making units (DMUs) with multiple inputs and outputs. It was first proposed by Charnes, Cooper and Rhodes in 1978 [25]. There are two important assumptions in the efficiency measurement of DEA [26]. One important decision to make when performing DEA is whether to use an input-orientation or output-orientation. The input-oriented model holds the current level of output constant and minimizes inputs, whereas an output-oriented model maximizes output while keeping the number of inputs constant. Another important theoretical hypothesis in DEA is whether the return to scale is constant or the returns to scale are variable. The first nonparametric models for efficiency estimation by Charnes, Cooper and Rhodes (CCR) assumed constant returns to scale (CRS) [25]. Later on, Banker, Charnes and Cooper (BCC) incorporated variable return to scale (VRS) to account for firms, which do not operate at their optimal scale [27]. At present, the choice of medical institutions in China depends mainly on patients and it is difficult to estimate the need for health services. Moreover, the government controls the input more than the output. Therefore, this paper chose to use the input-oriented BCC model to measure the annual TE from 2009 to 2017. The input-oriented BCC model is presented below:

$$minh=h\left(X_{0},Y_{0}\right)$$

(1) $$subject\;to\;\;\;\;\;\;\;\;\;\;hX_{0}-\overset{n}{\underset{j=1}{\sum }}\lambda_{\dot{J}}X_{j}\geq 0,\overset{n}{\underset{j=1}{\sum }}\lambda_{j}Y_{\dot{j}}\geq Y_{0},\overset{n}{\underset{\dot{j}=1}{\sum }}\lambda_{j}\;=1,\lambda_{\dot{J}}\geq 0,j=1,\ldots ,n$$

This relies on the fact that *h* ≥ 0 will be satisfied when the components of every *X_j_* and *Y_j_* are all nonnegative—as is the case for the observational data we are considering. This is a linear programming problem, the duality of which can be written as:

$$max\overset{s}{\underset{r=1}{\sum }}u_{r}y_{r0}-u_{0}$$

(2) $$\;subject\;to\;\;\;\;\;\;\;\;\;\;\;\;\;\overset{s}{\underset{r=1}{\sum }}u_{r}y_{rj}-\overset{m}{\underset{i=1}{\sum }}\nu_{i}x_{ij}-u_{0}\leq 0\;$$

(3) $$\overset{m}{\underset{i=1}{\sum }}\nu_{i}x_{i0}=1,u_{r,}\nu_{i}\geq 0$$

where $u_{0}$ is unconstrained in sign. If the $h\left(X_{0},Y_{0}\right)$ value is = 1, it is efficient; $h\left(X_{0},Y_{0}\right)$ value < 1, it is inefficient.

### 2.2. Malmquist Index Model 

The Malmquist index model is a non-parametric DEA model based on the productivity index research method first proposed by Malmquist. In 1982, Caves et al. first introduced the Malmquist Index (MI) as an efficiency index into the field of production analysis [28]. The Malmquist index model measures the change in total factor productivity change (TFP) of DMUs over time. The TFP is divided into two parts: Technical change (TECHCH) and technical efficiency change (EFFCH). The technical efficiency can be further decomposed into the product of pure technical efficiency change (PECH) and scale efficiency change (SECH). The input-oriented MI consists of four input-oriented distance functions that functionally represent multiple-input and multiple-output production techniques. They can be used to characterize efficiency because the distance function is the reciprocal of the technical efficiency measure proposed by Farrell [29]. The distance function is a natural way of modeling production boundaries because the deviations and offset from this boundary represent changes in efficiency and technology, respectively. This paper uses the Malmquist index model to measure the efficiency changes in Hunan Province from 2009 to 2017. The change in productivity between period *t* (base period) and period *t* + 1 is defined as:

(4) $$\begin{array}{ll} M_{0}\left(x_{t+1},y_{t+1},x_{t},y_{t}\right) & =\sqrt{\frac{D_{0}^{t}\left(x_{t+1},y_{t+1}\right)}{D_{0}^{t}\left(x_{t},y_{t}\right)}\times \frac{D_{0}^{t+1}\left(x_{t+1},y_{t+1}\right)}{D_{0}^{t+1}\left(x_{t},y_{t}\right)}}\\ & =\frac{D_{0}^{t}\left(x_{t+1},y_{t+1}\right)}{D_{0}^{t}\left(x_{t},y_{t}\right)}\sqrt{\frac{D_{0}^{t}\left(x_{t+1},y_{t+1}\right)}{D_{0}^{t+1}\left(x_{t+1},y_{t+1}\right)}\times \frac{D_{0}^{t}\left(x_{t},y_{t}\right)}{D_{0}^{t+1}\left(x_{t},y_{t}\right)}} \end{array}$$

where *M*_0_ is the input-oriented Malmquist productivity index, and *y* represents the output vector that can be produced using input vector x. Among them, EFFCH = $\frac{D_{0}^{t}\left(x_{t+1},y_{t+1}\right)}{D_{0}^{t}\left(x_{t},y_{t}\right)}$, TECHCH = $\sqrt{\frac{D_{0}^{t}\left(x_{t+1},y_{t+1}\right)}{D_{0}^{t+1}\left(x_{t+1},y_{t+1}\right)}\times \frac{D_{0}^{t}\left(x_{t},y_{t}\right)}{D_{0}^{t+1}\left(x_{t},y_{t}\right)}}$, respectively, indicate that efficiency improvement and technological progress occurred in the period t to t+1. Therefore, TFP can be expressed as follows: TFP = EFFCH × TECHCH. If the TFP > 1, indicates that the TFP of the t+1 period is higher than that of the t period; a TFP < 1, indicates a decrease in TFP; and TFP = 1 indicates that TFP is unchanged.

### 2.3. Tobit 

In this study, the TE obtained by DEA is the dependent variable, and the main factors affecting the TE of PHCIs in the county are explored by constructing regression models. Since the efficiency value measured by DEA ranges from [0, 1]; that is, the value less than 0 and greater than 1 is punctured, the dependent variable with punctured feature belongs to the restricted dependent variable. Estimating the regression parameters using a regression model based on the ordinary least squares method is likely to cause an estimation bias due to incomplete data. In view of this, Tobin first proposed the puncturing regression model, or the Tobit model, in 1958 [30]. The formula for the Tobit regression equation is the following:

(5) $$Y_{i}=\beta_{0}+\overset{n}{\underset{i=1}{\sum }}\beta_{i}X_{i}+\mu ,i=1,2,\ldots ,n$$

where $Y_{i}$ is the TE value, $X_{i}$ is the explanatory variable, and $\beta_{i}$ is the coefficient to be evaluated.

### 2.4. Data and Selection of Variables

Considering the integrity and availability of data, this study included 86 counties (county-level cities) in Hunan Province from 2009 to 2017; one county was not included because of partial missing data. The data were collected from the National Health Statistical Information Report System during the period 2009–2017, and the Hunan Statistics Yearbook.

In this paper, the input and output variables were selected by reviewing the relevant literature and based on the availability of data in the National Health Statistical Information Report System [16,31,32,33]. Regarding the input variables, both human resources and facilities were considered important for health care services. We selected the number of health technicians instead of human resources, and took the number of beds and the amount of equipment valued at more than 10,000 RMB to replace the facilities. The output indicators selected in this paper are the number of outpatients and emergency visits, and the number of discharged patients (refer to the standard explanation, Table 1).

In the Tobit regression analysis, influencing factors that affect PHCI’s efficiency indirectly were the total population, city level, per capita GDP, the proportion of health technicians, the proportion of beds (refer to the standard explanation, Table 1). 

The input and output variables were descriptively analyzed using SPSS 22.0 (International Business Machine, New York, America). The software DEAP 2.1 (University of New England, Armidale, Australia) was used to calculate the value of Technical Efficiency (TE) and Total Factor Productivity (TFP) and used the software STATA 12.0 (StataCorp LLC, Texas, TX, USA) for Tobit regression analysis.

## 3. Results

### 3.1. Descriptive Statistics of Inputs and Outputs

Table 2 shows the statistics of the input and output variables. From 2009 to 2017, the means of discharged patients, health technicians, beds and equipment valued at more than 10,000 RMB increased annually. During the same time, the number of outpatients and emergency visits has increased year by year from 2009 to 2013, and has decreased year by year from 2014 to 2015; after rising in 2016, it fell again.

**Figure 1:**
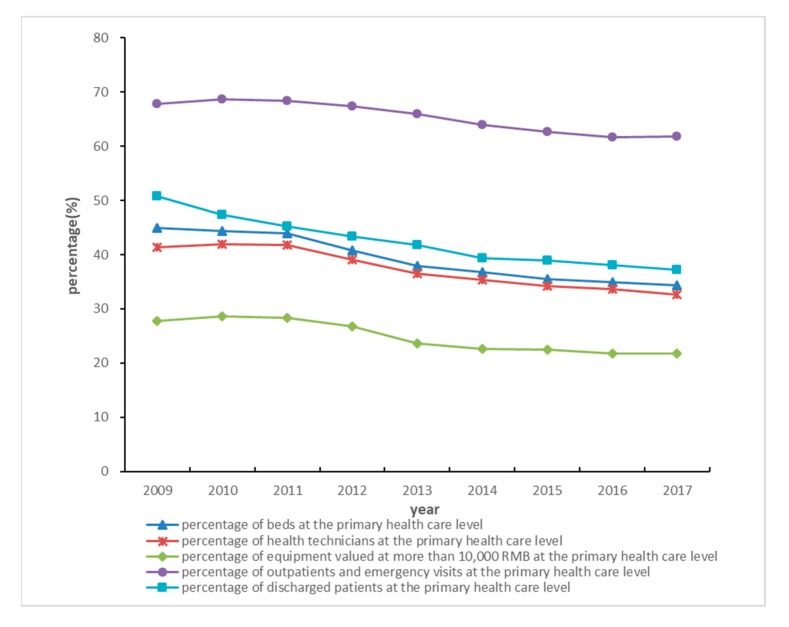
The percentage of inputs and outputs of primary health care institutions in 86 counties in Hunan Province from 2009 to 2017. Note: Percentage = sum of input (output) of primary health care institutions in 86 counties/sum of input (output) of medical institutions in 86 counties

As can be seen from the percentages of PHCI’s input and output indicators in all medical institutions, the outpatients and emergency visits, the discharged patients, health technicians, beds and equipment all showed a downward trend from 2009 to 2017. In addition to beds and discharged patients, other indicators increased significantly from 2009–2011, followed by a significant decline from 2012 to 2014, and a slowdown in 2015–2017 (Figure 1).

### 3.2. Results of the DEA Model 

As shown in Table 3, for the years 2009–2017, the TE of PHCIs in 86 counties in Hunan Province increased from 0.559 in 2009 to 0.754 in 2014, and increased slightly in 2017 after a small decline in 2016. Among the counties with effective TE, there were at least 5 (5.81%) counties in 2009, and the most in 2015 when there were 14 (16.28%).

In 2009–2017, there were about 5 (5.81%)–17 (19.77%) counties whose SE operated at the best level, with an SE score of 1.000. The average SE’s range was 0.813–0.928.

During the period from 2009 to 2015, TEvrs increased from 0.697 to 0.819. After a decrease in 2016, it increased again in 2017. The number of effective counties fluctuated between 16 and 24. 

### 3.3. Results of the Malmquist Index Model

**Figure 2:**
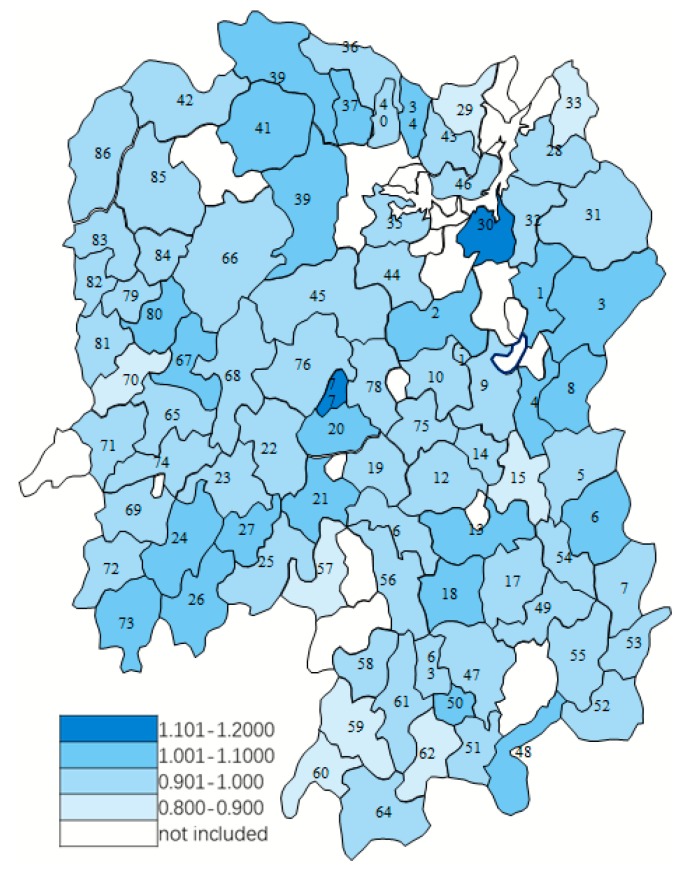
Distribution of factor productivity change (TFP) in 86 counties (county-level cities) from 2009 to 2017.

The Malmquist index model was applied to analyses on the changes in productivity over the 2009–2017 period, and the year 2009 was taken as the technical reference. Table 4 presents the Malmquist index summary of annual geometric means. On average, TFP decreased by 3.1%, among which, TECHCH decreased by 6.5% and EFFCH increased slightly by 3.7%. During the period 2009–2017, 24 (27.91%) counties had TFP score greater than 1, indicating growth in TFP; 61 (70.93%) counties had TFP score less than 1, indicating a deterioration in TFP. Figure 2 shows the distribution of TFP in Hunan Province. The most efficient counties are Xiangyin County (30) and Lengshuijiang City (77). The TFP of each county is shown in Table A1, Appendix A.

### 3.4. Results of Tobit Regression

In this article, Tobit regression was used to analyze the influencing factors of TE during 2009–2017. The results are presented in Table 5. In order to reduce heteroscedasticity, we have performed a logarithmic conversion on the two independent variables of population and per capita GDP. Regarding environmental factors, Ln (total population) (*p* < 0.001), city level (*p* < 0.001), the proportion of health technicians (*p* < 0.001), the proportion of beds (*p* = 0.002) exhibited significance, indicating that they are significant in influencing the efficiency of PHCIs. However, Ln (per capita GDP) (*p* = 0.977) was statistically insignificant.

## 4. Discussion

Since the new health-care reform in 2009, the government has invested a lot in monetary, equipment, buildings and health technicians in PHCIs, but the growth of these resources has not brought about an increase in the use of PHCIs, residents’ choice of health care services still tends to tertiary hospitals [8,35]. In May 2019, the National Health Commission of People’s Republic of China issued a policy document requiring the overall performance of the county-level health system and the ability of PHCIs to be improved, and the visit rate of PHCIs in the county is required to reach 65% [36]. However, in recent years, the visit rate of PHCIs has been declining. This paper can provide a reference for improving the efficiency of PHCIs by studying the efficiency of PHCIs in 86 counties of Hunan Province and their influencing factors.

The BCC model is a method used to analyze the efficiency of a single period. This study used the BCC model to measure the annual efficiency of PHCIs in Hunan Province each year from 2009 to 2017. It was found that the average TE of PHCIs in Hunan Province in the past nine years was lower than 0.8 and most counties were ineffective, which indicates that the efficiency of PHCIs needs to be improved. Besides, the TE and the counties with effective TE both increased from 2009 to 2015 but declined in 2016. While we cannot know the trend of efficiency after 2018, a potential explanation is that the dissolution and consolidation of PHCIs in Hunan Province during 2015–2016 that affected the continuity of PHC and according to the general choice of patients and increasingly convenient traffic conditions, patients who previously went to the revoked PHCIs may not go to the retained PHCIs, but are more likely to a secondary or tertiary hospital [37,38,39]. Compared to SE, TEvrs was lower, which indicates that while improving the scale of PHCIs, we must pay more attention to improving efficiency by improving the medical technology of PHCIs. Compared with other studies using the same method, the average TE of PHCIs in Hunan Province was higher than that in the Community Health Centers of Jiangsu, which is 0.15–0.40 [40], but lower than China’s total medical services efficiency, which is 0.904 [41]. This difference may be due to different regions or research subjects. 

The Malmquist Index Model measures the dynamic efficiency change of the DMU over multiple periods. The results of the Malmquist Index Model verified that PHCIs in Hunan experienced a significant decline in TFP from 2009 to 2017, which was consistent with the results of Zhang et al. [23]. The trend of TECHCH and TFP was consistent, so it can be considered that the change of TFP was mainly affected by TECHCH changes, which is consistent with the findings of Leng et al. [4]. Chen et al. pointed out that technological progress determines the key factors of the TFP of community health services in China [42]. The main reason for the low TECHCH is the lack of excellent health technicians, and the underutilization of medical equipment in PHCIs. 

Some studies indicate that health resources in areas with low economic levels can be better utilized, which may increase efficiency [18,43,44]. In this study, the Tobit regression results indicated that the impact of the per capita GDP on the growth of TE of PHCIs is not significant. This is consistent with the findings of Leng et al. who found that this is mainly due to the guiding role of government policies and the implementation of health-care reform policies that have narrowed the gap in regional health resource allocation [4]. Therefore, the level of economic between regions has a small impact on TE.

The positive impact of the total population on TE in this study is consistent with the findings of Tan et al. [45]. The number of permanent residents at the end of the year reflects the potential health care service needs in the region, which will affect the efficiency of the hospital. In addition, the increase in China’s population is accompanied by the implementation of the two-child policy and the development of an aging population, which has also increased the demand and utilization of health services [46,47]. However, research by Chen et al. suggested that a large population will increase the burden on the hospital and make health care service inefficient, and this may be caused by different research objects [48]. 

This study shows that the city level has a negative impact on TE. At the same time, Zhang et al. showed that the impact of urbanization on the efficiency of PHCIs in the rural and urban areas is different [49]. With the advancement of urbanization in China, the number of poor people has decreased. Zhang et al. showed that wealthy people are more likely to use hospitals with adequate resources, and poorer people are more likely to use poorly funded PHCIs for health care services [50]. Meanwhile, due to the relatively concentrated population of urbanization and the convenience of modern transportation, which has further improved the geographic accessibility of health care services, residents may be more inclined to seek higher quality health care services at secondary or tertiary hospitals, which has a negative impact on the efficiency of PHCIs [51].

While the new health-care reform has increased the number of health technicians and beds in PHCIs, TE has decreased as their proportion increased. In the long run, a lack of qualified health technicians and inadequate use of medical equipment may reduce technical efficiency [52]. PHCIs still have problems such as severe maldistribution of quality human resources, low education of health technicians and the detrimental elements of health-care reform policies [53]. These problems have led to a lack of patients’ trust in the quality of PHC. Therefore, patients’ demands for high-quality health care services make the gradient design of the health insurance reimbursement rate insufficient to guide patients to PHCIs for treatment [20,54]. Meanwhile, due to the small development space, low wages and lack of sufficient performance incentives of PHCIs, the enthusiasm of health technicians has been affected, limiting the improvement of PHCIs’ efficiency [5,55,56]. After the implementation of the new health-care reform, the government provided a lot of advanced medical equipment for PHCIs, but the health technicians in the PHCIs lacked operating skills and could not use the equipment well [57,58]. As a result, a large amount of equipment and beds were idle, which hindered PHCIs in improving efficiency.

This study conducted a horizontal and vertical analysis of the efficiency of PHCIs from 2009 to 2017 from the perspective of the county, and explored its influencing factors. However, there are some limitations. First, the input-output variables and influencing factors in this paper were selected based on the literature and the availability of data, which may lead to deviations in the research results. Second, the TE of PHCIs may be affected by other factors not considered in this article, such as government health expenditure, the education level of the residents, life expectancy, et al. Third, bias adjustments of TE and TFP scores were not carried out due to the limitation of the basic DEA approach. Fourth, due to the availability of data, we only use the data from 2009 to 2017 for analysis, which cannot be compared with the efficiency of PHCIs in other periods. Despite these limitations, this study can be considered a useful attempt to measure the TE and TFP of PHCIs at the county level in China.

## 5. Conclusions 

In this study, we utilized a panel dataset of 86 county-level units of Hunan during the period 2009–2017 to describe the allocation of health resources and the provision of health care services in PHCIs, to compare the efficiency of the regional primary health care systems, and to explore the determinants of TE. The main conclusions of this study were as follows: Firstly, the efficiency of PHCIs in Hunan Province during 2009–2017 was low, and most counties are inefficient. Secondly, the results of the Malmquist index showed that the PHCIs in Hunan Province experienced significant decline in TFP from 2009 to 2017, which mainly resulted from the low TECHCH. Third, the factors affecting the TE of PHCIs are the total population, city level, the proportion of health technicians and the proportion of beds in PHCIs. 

According to the conclusions, we propose the following suggestions: First, we need to improve the medical technology skills of health technicians, strengthening the training of clinical skills, health management skills and equipment operation skills of health technicians, and creating policies on job title and continuing education could help to attract excellent health technicians to work in PHCIs. Second, improving the performance policy of PHCIs would allow PHCIs to independently determine internal performance wages and distribution methods, and increase the proportion of incentive performance wages on the premise of guaranteeing the payment of basic wages, thereby enhancing the enthusiasm of health technicians. Third, improving the payment policy of medical insurance funds, by increasing the total annual budgetary payment of PHCIs in the county medical insurance fund. The increase should be higher than the evaluation increase of the total annual budget payment of local medical institutions, and should shape the medical insurance payment policy to guide residents who have common diseases or frequently occurring diseases to go to PHCIs for health care service.

## Figures and Tables

**Table 1:** Definitions of input, output and influencing factors [34].

Category	Variable	Definition	Unit
Input	Number of health technicians	Health technicians include licensed physicians, licensed assistant physicians, registered nurses, pharmacists, laboratory technicians, radiologists, health supervisors and interns.	person
	Number of beds	The number of beds refers to the number of fixed beds at the end of the year, including regular beds, simple beds, monitoring beds, extra beds for more than half a year, beds being disinfected and repaired and beds disabled due to expansion or overhaul.	quantity
	The amount of equipment valued at more than 10,000 RMB	The amount of equipment of more than 10,000 RMB	set
Output	Number of outpatients and emergency visits	The number of patients attending outpatient and emergency diagnostic services	person
	Number of discharged patients	The number of people discharged after hospitalization at the end of each year	person
Influencing factor	The total population	The population of this study refers to the population of each county at 24 o’clock on December 31 each year.	10,000 persons
	City level	The proportion of the urban population in each county to the total population	%
	Per capita GDP	Per capita GDP of each county	RMB
	The proportion of health technicians in PHCIs	The proportion of health technicians in PHCIs to the number of health technicians in all medical institutions in the county	%
	The proportion of beds in PHCIs	The proportion of beds in PHCIs to the number of beds in all medical institutions in the county	%

**Table 2:** Descriptive statistics of inputs and outputs.

Year	Measure	The Number of Outpatients and Emergency Visits	The Number of Discharged Patients	The Number of Health Technicians	The Number of Beds	The Amount of Equipment Valued at More than 10,000 RMB
2009	Mean	962,707.21	29,345.60	708.23	676.64	125.92
	SD	724,110.69	18,350.91	425.51	564.43	107.41
	Min	110,131.00	4518.00	116.00	150.00	11.00
	Max	3,953,181.00	114,585.00	1646.00	4577.00	811.00
2010	Mean	1,066,902.41	28,688.70	758.60	693.65	154.16
	SD	930,754.12	18,855.63	455.23	415.77	141.45
	Min	111,788.00	2850.00	139.00	157.00	17.00
	Max	7,381,078.00	120,918.00	2016.00	2769.00	1131.00
2011	Mean	1,091,901.15	31,257.56	799.17	779.91	172.35
	SD	744,620.69	2161.58	553.31	600.63	154.24
	Min	111,287.00	3448.00	111.00	144.00	26.00
	Max	4,132,915.00	129,426.00	3408.00	4596.00	1196.00
2012	Mean	1,141,418.03	35,452.37	776.38	835.05	187.45
	SD	729,038.34	26,541.61	488.28	640.10	183.01
	Min	122,637.00	3173.00	81.00	141.00	23.00
	Max	3,780,418.00	161,004.00	2712.00	4677.00	1497.00
2013	Mean	1,169,242.55	37037.21	789.40	829.48	197.62
	SD	732,494.83	28,594.02	492.72	519.22	198.05
	Min	99,575.00	4113.00	113.00	156.00	20.00
	Max	3,802,118.00	198,410.00	2908.00	3353.00	1657.00
2014	Mean	1,138,600.08	36,502.15	801.26	900.16	216.56
	SD	695,198.60	29,741.37	477.59	573.65	196.28
	Min	88,394.00	3924.00	142.00	165.00	22.00
	Max	3,770,194.00	210,175.00	2947.00	3557.00	1544.00
2015	Mean	1,125,796.98	38,390.83	850.12	1005.55	236.78
	SD	676,580.06	30,452.48	543.67	668.92	229.35
	Min	79,844.00	3,138.00	137.00	169.00	25.00
	Max	3,294,833.00	206,940.00	3032.00	3775.00	1886.00
2016	Mean	1,130,715.92	40,448.90	882.20	1052.35	261.60
	SD	704,462.45	32,539.49	541.39	699.29	254.37
	Min	101,379.00	3489.00	118.00	206.00	21.00
	Max	3,564,319.00	214,586.00	3115.00	3900.00	2068.00
2017	Mean	1,095,871.95	41,745.28	909.79	1106.35	287.33
	SD	708,005.71	36,279.66	548.31	729.51	323.38
	Min	86,996.00	1473.00	137.00	180.00	36.00
	Max	3,311,100.00	247,733.00	3238.00	4090.00	2756.00

**Table 3:** The efficiency of Primary Health Care Institutions (PHCIs) in the counties of Hunan Province from 2009 to 2017 (input-oriented Banker, Charnes and Cooper (BCC) model).

Year	TE ^1^		TEvrs ^2^		SE ^3^	
	Mean	Number of Efficient Counties	Mean	Number of Efficient Counties	Mean	Number of Efficient Counties
2009	0.559	5(5.81%)	0.697	16(18.60%)	0.813	5(5.81%)
2010	0.656	8(9.30%)	0.760	18(20.93%)	0.869	9(10.47%)
2011	0.699	8(9.30%)	0.789	20(23.26%)	0.889	9(10.47%)
2012	0.737	11(12.79%)	0.798	19(22.09%)	0.925	12(13.95%)
2013	0.747	12(13.95%)	0.811	22(25.58%)	0.920	17(19.77%)
2014	0.754	13(15.12%)	0.813	22(25.58%)	0.928	16(18.60%)
2015	0.754	14(16.28%)	0.819	24(27.91%)	0.922	16(18.60%)
2016	0.690	9(10.47%)	0.780	14(16.28%)	0.888	9(10.47%)
2017	0.738	11(12.79%)	0.822	21(24.42%)	0.898	11(12.79%)

^1^ TE: Technical efficiency; ^2^ TEvrs: Technical efficiency from the variable return to scale Data Envelopment Analysis (VRS DEA); ^3^ SE: Scale efficiency = TE/TEvrs.

**Table 4:** Results of the Malmquist index model (input-oriented).

Year	EFFCH ^1^	TECHCH ^2^	PECH ^3^	SECH ^4^	TFP ^5^
2010	1.186	0.766	1.103	1.075	0.909
2011	1.075	0.917	1.043	1.030	0.986
2012	1.061	0.995	1.014	1.046	1.055
2013	1.015	0.990	1.018	0.997	1.005
2014	1.010	0.908	1.005	1.005	0.917
2015	0.998	0.975	1.005	0.993	0.973
2016	0.910	1.072	0.948	0.960	0.976
2017	1.060	0.886	1.056	1.004	0.940
mean	1.037	0.935	1.023	1.013	0.969
Frequency distribution					
>1	66 (76.74%)	0 (0.00%)	60 (69.77%)	60 (69.77%)	24 (27.91%)
=1	1 (1.16%)	0 (0.00%)	10 (11.63%)	5 (5.81%)	1 (1.16%)
<1	19 (22.09%)	86 (100.00%)	16 (18.60%)	21 (24.42%)	61 (70.93%)

^1^ EFFCH: Technical efficiency change; ^2^ TECHCH: Technical change; ^3^ PECH: Pure technical efficiency change; ^4^ SECH: Scale efficiency change; ^5^ TFP: Total factor productivity change.

**Table 5:** Results of Tobit regression (*N* = 774).

Variable	Coefficient	SE	T-Ratio	*p*	95% CI
Ln (the total population)	0.104	0.014	7.36	< 0.001 ***	(0.076–0.132)
city level	−0.456	0.117	−3.89	< 0.001 ***	(−0.686 to −0.226)
Ln (per capita GDP)	0.001	0.019	0.03	0.977	(−0.037–0.038)
the proportion of health technicians	−0.462	0.087	−5.31	< 0.001 ***	(−0.633 to −0.291)
the proportion of beds	−0.181	0.058	−3.12	0.002 **	(−0.295 to −0.067)
Constant	0.699	0.168	4.15	< 0.001 ***	(0.368–1.029)
Sigma	0.200	0.006			(0.189–0.211)
Log likelihood	24.141				
$\chi^{2}$	77.750				
Probability >$\;\chi^{2}$	< 0.001 ***				

Note: *** *p* < 0.001; ** *p* < 0.01.

## Abbreviations

UHC	Universal Health Coverage
WHO	World Health Organization
PHC	Primary Health Care
PHCIs	Primary Health Care Institutions
DEA	Data Envelopment Analysis
TE	Technical Efficiency
DMUs	decision-making units
CCR	Charnes, Cooper, and Rhodes
BCC	Banker, Charnes, and Cooper
CRS	constant returns to scale
VRS	variable return to scale
MI	the Malmquist Index
TFP	total factor productivity change
TECHCH	technical change
EFFCH	technical efficiency change
PECH	pure technical efficiency change
SECH	scale efficiency change

## Appendix A

**Table A1:** TFP in 86 counties (county-level cities) in Hunan Province from 2009 to 2017.

Number	Counties	Efficiency
1	Changsha County	1.049
2	Ningxiang County	1.019
3	Liuyang City	1.019
4	Zhuzhou County	1.012
5	You County	0.985
6	Chaling County	1.012
7	Yanling County	0.985
8	Liling County	1.007
9	Xiangtan County	0.936
10	Xiangxiang City	0.933
11	Shaoshan City	0.922
12	Hengyang County	0.961
13	Hengnan County	1.001
14	Hengshan County	0.913
15	Hengdong County	0.880
16	Qidong County	0.939
17	Leiyang City	0.956
18	Changning City	1.030
19	Shaodong County	0.964
20	Xinshao County	1.001
21	Shaoyang County	1.014
22	Longhui County	0.969
23	Dongkou County	0.964
24	Suining County	1.001
25	Xinning County	0.959
26	Chengbu County	1.018
27	Wugang City	0.947
28	Yueyang County	0.929
29	Huarong County	0.856
30	Xiangyin County	1.112
31	Pingjiang County	0.999
32	Miluo City	0.916
33	Linxiang City	0.821
34	Anxiang County	1.006
35	Hanshou County	0.973
36	Li County	0.981
37	Linli County	1.019
38	Taoyuan County	1.016
39	Shimen County	1.016
40	Jinshi City	0.983
41	Cili County	1.028
42	Sanzhi County	0.950
43	Nan County	0.967
44	Taojiang County	0.972
45	Anhua County	0.963
46	Yuanjiang City	0.986
47	Guiyang County	0.939
48	Yizhang County	1.016
49	Yongxing County	0.969
50	Jiahe County	1.032
51	Linwu County	0.989
52	Rucheng County	0.976
53	Guidong County	0.917
54	Anren County	0.918
55	Zixing County	0.955
56	Qiyang County	0.980
57	Dongan County	0.888
58	Shuangpai County	0.954
59	Dao County	0.877
60	Jiangyong County	0.889
61	Ningyuan County	0.919
62	Lanshan County	0.885
63	Xintian County	0.918
64	Jianghua County	0.954
65	Zhongfang County	0.985
66	Yuanling County	0.979
67	Chenxi County	1.036
68	Xupu County	0.999
69	Huitong County	0.978
70	Mayang County	0.890
71	Zhijiang County	0.966
72	Jingzhou County	0.946
73	Tongdao County	1.045
74	Hongjiang City	0.929
75	Shuangfeng County	0.942
76	Xinhua County	0.973
77	Lengshuijiang City	1.143
78	Lianyuan City	1.000
79	Jishou City	0.907
80	Luxi County	1.051
81	Fenghuang County	0.982
82	Huayuan County	0.996
83	Baojing County	0.906
84	Guzhang County	0.954
85	Yongshun County	0.997
86	Longshan County	0.994
